# Enhancing Inhibition-Induced Plasticity in Tinnitus – Spectral Energy Contrasts in Tailor-Made Notched Music Matter

**DOI:** 10.1371/journal.pone.0126494

**Published:** 2015-05-07

**Authors:** Alwina Stein, Alva Engell, Pia Lau, Robert Wunderlich, Markus Junghoefer, Andreas Wollbrink, Maximilian Bruchmann, Claudia Rudack, Christo Pantev

**Affiliations:** 1 Institute for Biomagnetism and Biosignalanalysis, University of Muenster, Muenster, Germany; 2 Department of Otolaryngology, University Hospital, Muenster, Germany; Kyoto University, JAPAN

## Abstract

Chronic tinnitus seems to be caused by reduced inhibition among frequency selective neurons in the auditory cortex. One possibility to reduce tinnitus perception is to induce inhibition onto over-activated neurons representing the tinnitus frequency via tailor-made notched music (TMNM). Since lateral inhibition is modifiable by spectral energy contrasts, the question arises if the effects of inhibition-induced plasticity can be enhanced by introducing increased spectral energy contrasts (ISEC) in TMNM. Eighteen participants suffering from chronic tonal tinnitus, pseudo randomly assigned to either a classical TMNM or an ISEC-TMNM group, listened to notched music for three hours on three consecutive days. The music was filtered for both groups by introducing a notch filter centered at the individual tinnitus frequency. For the ISEC-TMNM group a frequency bandwidth of 3/8 octaves on each side of the notch was amplified, additionally, by about 20 dB. Before and after each music exposure, participants rated their subjectively perceived tinnitus loudness on a visual analog scale. During the magnetoencephalographic recordings, participants were stimulated with either a reference tone of 500 Hz or a test tone with a carrier frequency representing the individual tinnitus pitch. Perceived tinnitus loudness was significantly reduced after TMNM exposure, though TMNM type did not influence the loudness ratings. Tinnitus related neural activity in the N1m time window and in the so called tinnitus network comprising temporal, parietal and frontal regions was reduced after TMNM exposure. The ISEC-TMNM group revealed even enhanced inhibition-induced plasticity in a temporal and a frontal cortical area. Overall, inhibition of tinnitus related neural activity could be strengthened in people affected with tinnitus by increasing spectral energy contrast in TMNM, confirming the concepts of inhibition-induced plasticity via TMNM and spectral energy contrasts.

## Introduction

The human nervous system changes dynamically with experience. This phenomenon, named plasticity, was first described by James in 1890 [1] and is still of enormous research interest. The permanent structural and functional adaptation to external demands is not only restricted to childhood, but remains in adulthood as well as in the aging brain [2,3]. One prerequisite for the functionality of brain plasticity is the balance between excitatory and inhibitory neural activation [4]. If this balance gets disturbed, dysfunctionalities such as developmental intellectual disabilities [5], autism [4] or phantom perceptions [6–8] can appear.

Tinnitus, a syndrome assumed to derive from imbalanced excitation and inhibition in the auditory cortex [9–11], is another example of dysfunctional plasticity. It is defined as the perception of a sound without an external source [12]. The perception is described as one or several sounds of different quality (e.g. beeping, pulsing, hissing etc.), though tonal tinnitus (whistling or ringing sound) is most prominent [13,14]. With a prevalence rate of 10–15% in industrialized countries [15,16] and a 10-year cumulative incidence rate of 12.7% for people aged between 48 and 92 years [17], chronic tinnitus is a quite common, though hardly treatable [18,19] syndrome.

One major cause for chronic tinnitus is reduced inhibition in the auditory cortex [12,20,21], triggered by a certain degree of hearing loss. Yet, this hearing loss may not always be detectable with conventional audiometric procedures [12,22]. The hearing loss causes a deafferentation of auditory neurons coding the corresponding region in the auditory cortex [23]. The deafferented neurons in turn cannot spread lateral inhibition onto neurons coding the audiometric edge. Due to neural plasticity, the system adapts to the resulting lack of reciprocal and lateral inhibition [24,25] by tonotopically reorganizing its cortical structure [9,26]. The maladaptive plasticity is reflected in reduced inhibition of neurons coding the audiometric edge, increased synchronous firing as well as enhanced spontaneous firing rates in the reorganized region of the auditory cortex [27,28]. Finally, over-activated neurons in the reorganized region seem to be responsible for the tinnitus perception [11]. Overall, tinnitus appears as result of dysfunctional neural plasticity and is thus referred to as a “plasticity disorder”, which Möller describes as a disorder “where the symptoms are caused by plastic changes that are not beneficial to an individual person” [29].

Since tinnitus seems to be caused by reduced inhibition, a possible treatment strategy is the induction of inhibition onto the over-activated neurons representing the tinnitus frequency (TF) [30]. The stimulation with notch-filtered sounds provides a good possibility for doing this. Pantev et al. [31] and later Okamoto and colleagues presented notch-filtered noise as a masking stimulus to normal hearing subjects [32,33]. The processing of a subsequent stimulus with a carrier frequency of the notch center frequency was inhibited as compared to pure habituation effects by white noise or stop-band noise masking. The decline of auditory evoked responses 100 ms after stimulus onset (N1m) was discussed as a mechanism of lateral inhibition, which was confirmed in following studies [34,35].

The direct effect of lateral inhibition decreases as a function of reduced masker duration [36] and with the temporal distance between masker and test stimulus; the greater the interval between notched noise and test stimulus, the smaller the effect of lateral inhibition [34]. However, a massive induction of lateral inhibition over an expanded period of time also results in reduced processing of the formerly inhibited stimulus. Pantev and coworkers, for instance, presented notched music to normal hearing subjects for three hours on three consecutive days [37]. The result was a reduced processing of frequency bandwidths within the notch after music exposure, which was discussed in terms of plasticity in the auditory cortex due to induction of lateral inhibition. Since plasticity is defined as dynamic change of our nervous system caused by experience, a prolonged reduction of stimulus processing due to the induction of lateral inhibition of neurons coding this stimulus is what we call inhibition-induced plasticity.

The mechanisms of inhibition-induced plasticity were applied as a treatment strategy against tinnitus by inducing inhibition onto neurons coding the TF. Okamoto et al. presented music with a notch filter centered at the individual TF to participants affected by tonal tinnitus for two hours each day over a period of one year (tailor-made notched music training, TMNMT) [38]. They demonstrated that tinnitus related neural activity in the auditory cortex as well as subjectively perceived tinnitus loudness was significantly reduced as compared to a placebo and a monitoring group. A similar effect was found in another treatment study of shorter duration [39].

The question arises, if the above mentioned treatment effects of inhibition-induced plasticity could be further enhanced. Lateral inhibition was found to be modifiable by spectral energy contrasts variation in notched noise [40]. More precisely, it could be demonstrated that an amplification of the edge frequency bands around the notch resulted in the greatest lateral inhibition of neurons coding the center frequency of the notch. Therefore, the edge frequency bands around the notch seem to be most relevant to induce lateral inhibition onto the center frequency of the notch, which was confirmed in animal data [35]. If lateral inhibitory effects were transferrable to inhibition-induced plasticity, an enhancement of inhibition-induced plasticity should be achieved by amplifying the edge-frequency bands around the notch in tailor-made notched music (TMNM). Furthermore, since over-activated neurons seem to be responsible for the tinnitus perception, an increased inhibition of these neurons should result in a stronger reduction of the subjectively perceived tinnitus loudness.

Recently, we demonstrated inhibition-induced plasticity in participants affected with tinnitus [41]. We adapted the above mentioned study design of Pantev and colleagues [37] by exposing participants with chronic tinnitus to three hours of TMNM on three consecutive days. The result was a reduced tinnitus loudness perception as well as reduced tinnitus related neural activity after TMNM exposure. Interestingly, this reduction was not only visible in the auditory cortex, but rather in all nodes of the so called tinnitus network [42–44] comprising temporal, parietal and frontal regions. Within the scope of the just described study, we additionally measured a group of subjects suffering from chronic tinnitus which listened to TMNM with further increased spectral energy contrasts (ISEC-TMNM).

The current study compared inhibition-induced plasticity by classical TMNM versus ISEC-TMNM. Here we hypothesized:

1. Overall, neural activity evoked by the TF should be reduced after TMNM exposure in a tinnitus network comprising temporal, parietal and frontal regions in the N1m time window.
2. Reduction of neural activity after music exposure should be greater in the ISEC-TMNM group as compared to the classical TMNM group within the N1m time window.
3. Overall, subjectively perceived tinnitus loudness should be reduced after TMNM exposure.
4. Tinnitus loudness reduction after TMNM exposure should be greater in the ISEC-TMNM group as compared to the classical TMNM group.

The aim of this study was therefore twofold. On the one hand we wanted to test our concept of inhibition-induced plasticity by manipulating the degree of lateral inhibition, hereby testing the transferability of effects of lateral inhibition on inhibition-induced plasticity. Additionally, we wanted to improve the tailor-made notched music training (TMNMT), by transferring basic research into clinical practice.

## Materials and Methods

### Subjects

Eighteen subjects (mean age = 47.06, range from 26–63 years; 12 male) were recruited from a database of the Institute for Biomagnetism and Biosignalanalysis of the University of Muenster. All subjects in the data base previously participated in a diagnostic screening including an anamnestic interview, an ear examination conducted by an otolaryngologist, a hearing test and a TF determination. The hearing test and the TF determination were done using an appropriate audiometer (MADSEN Astera, Denmark) and calibrated headphones (Sennheiser HDA 200). TF determination consisted of three steps including a presentation of seven predefined sine tones (between 1 and 12.5 kHz), a two-forced choice task until only one frequency remained and an octave confusion test (see [45] for a detailed description).

Subjects were included in the current study, if they fulfilled the following criteria: 1. Tonal tinnitus perception for more than three months (= chronic tinnitus). 2. TF between 1.5 kHz and 8.5 kHz. 3. Maximal hearing loss of 65 dB in a range of ½ octaves below or above the individual TF. 4. No acute or chronic otological, neurological or psychiatric disorders. 5. No drug abuse. 6. Subjects were right handed. These criteria were defined to ensure an optimal effect of TMNM. A hearing level of equal or less than 65 dB should guarantee an adequate perception of TMNM. The tinnitus pitch limitation was defined due to limitations in our magnetoencephalographic system (MEG). However, according to the data and literature available, we cannot state which exact levels of hearing loss or tinnitus pitch might be limitations of the general effectiveness of TMNM.

### Ethics Statement

The study was conducted according to the Declaration of Helsinki and the study protocol was approved by the ethics committee of the Medical Faculty of the University of Muenster. Subjects were fully informed about the study’s content and gave written consent before participating.

### Procedure and Stimuli

**Fig 1:**
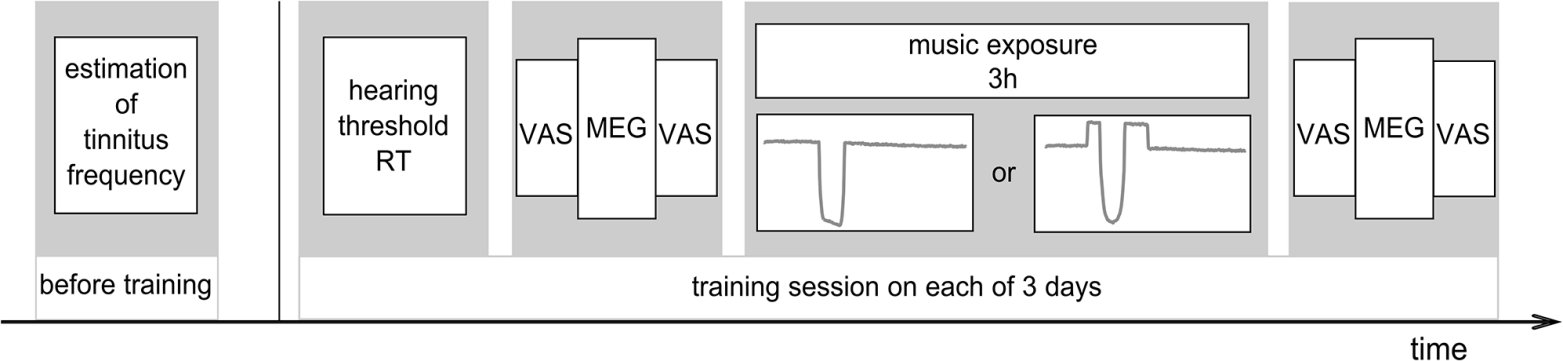
Study procedure. Tinnitus frequency was estimated before beginning of the training. The training session consisted of a hearing threshold determination for the reference tone of 500 Hz (RT). Ratings on the visual analog scale (VAS) were done before and after MEG measurements. Classical or ISEC tailor-made notched music (TMNM) was presented for 3 hours in between two VAS/MEG/VAS sessions. The training session was repeated on the next two days.

The study design is depicted in Fig 1. The procedure started with a TF determination to ensure an updated TF. Subjects participated in the music training, as described below, for three hours on three consecutive days. They were pseudo randomly assigned to either a group receiving classical TMNM or a group receiving ISEC-TMNM treatment. Heterogeneity between groups was minimized as controlled by independent *t*-tests for the factors (i) age, (ii) TF, (iii) hearing loss at TF and (iv) maximum hearing loss ½ octaves above or below TF. The values for the TF are naturally logarithmic and were transformed into octaves (with 1000 Hz as reference frequency) to achieve a linear distance between values, making a test of group homogeneity possible. The transformation formula was: Log2*(Hz/1000). Before and after each training session, auditory evoked fields were recorded by means MEG recordings.

**Fig 2:**
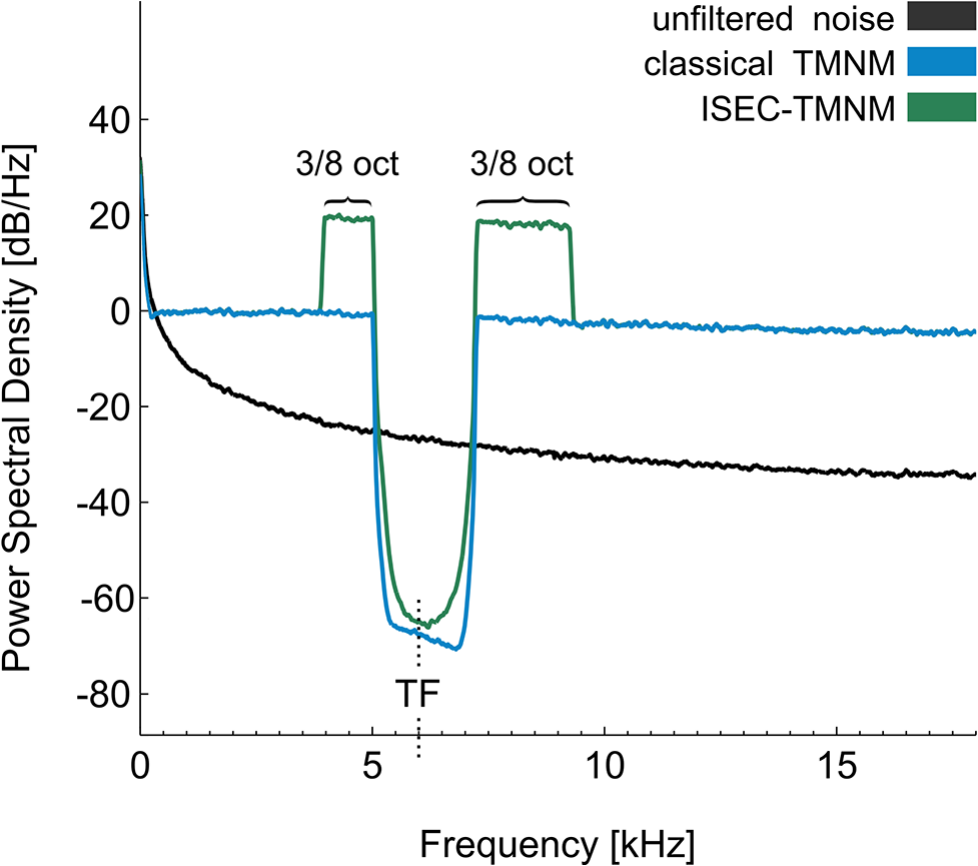
Power spectral density of Brownian noise filtered with both procedures. Brownian noise was chosen to illustrate the filter procedure, as it resembles the typical energy spectrum of music (lower energy in higher frequencies, 1/f behavior). Black line: frequency spectrum of Brownian noise with 3 s duration. Blue line: frequency spectrum of 3 s of Brownian noise filtered with the applied online filter for classical tailor-made notched music (TMNM). First, spectral energy was re-distributed from low to high frequency ranges (“flattening”). Second, a frequency band of 1/2 octave width centered at 6 kHz was removed from the energy spectrum of Brownian noise (classical TMNM). Green line: frequency spectrum of 3 s of Brownian noise filtered with the applied online filter for increased spectral energy contrast TMNM (ISEC-TMNM). In addition to the filtering procedure of classical TMNM, 3/8 octaves bandwidth of the edge frequency bands around the notch were amplified by 20 dB.

Training session: Subjects were seated in a silent room and were supplied with an MP3 player. Before participation, subjects provided CDs and digital music files with approximately three hours of their favorite music. This music was filtered online during the training session. The filtering procedure differed between the two groups in the following manner: in the classical TMNM group, music was modified on-line in two steps. First, music energy spectrum was equalized by redistributing energy of lower frequencies to higher frequencies, which is also called “flattening” [39]. This procedure compensates the energy decrement by a factor of 1/f which typically characterizes music frequency spectra in order to increase lateral inhibitory mechanisms from higher frequency areas. In a second step, a notch filter with ½ octaves bandwidth centered at the individual TF was applied to the music. In the ISEC-TMNM group, the filter procedure included both steps of the classical TMNM group. However, in addition frequency bandwidths of 3/8 octaves on each side of the notch were amplified by 20 dB. The bandwidth of 3/8 octaves was chosen, since it could be demonstrated that an energy amplification for this frequency band around the notch results in the greatest lateral inhibition of neurons coding the center frequency of the notch [40]. We chose an amplification of only 20 dB in the edge frequency bands around the notch, as a stronger amplification would render the music experience less pleasant. An illustration of power spectra as a result of applying both types of filter procedures to Brownian noise are shown in Fig 2. Participants were instructed to listen to the music at a comfortable loudness. During the training session, they were allowed to read or surf the internet and their alertness was checked every hour by a researcher. To ensure that, in the auditory domain, participants were exclusively stimulated by TMNM, they were asked to refrain from any verbal interaction and necessary communication was conducted in written form. Participants listened to TMNM for three hours until they were seated in the MEG shielded room. Headphones were removed in the MEG chamber shortly before MEG stimulation started.

MEG stimulation: In the MEG, subjects were stimulated with a reference tone of 500 Hz (RT) and a tinnitus tone (TT), representing their individual TF. Both stimuli consisted of a combined sound stimulus of 1000 ms duration. The first 300 ms consisted of a sine tone; the last 700 ms were 40 Hz amplitude modulated. We demonstrated previously that only the N1m response is modulated by spectral energy contrasts, not the ASSR evoked by the 40 Hz amplitude modulated tone [40]. Also Teismann and his colleagues [39] could not find an ASSR decrease after five days of TMNM exposure and argued that plasticity changes in primary auditory cortex, as depicted by ASSR differences, might need more time to develop. Therefore, we will focus in this study only on the sine tone evoking the N1m response (also cf. [41]). Sound was delivered through silicon plastic tubes (60 cm length, inner diameter of 5 mm) with silicon earpiece endings matching the subject’s ears. Both stimuli were presented binaurally at an intensity of 45 dB SL. Before stimulation, hearing threshold for the RT was determined with a clinical speech audiometer (Midimate 622, Madsen, Denmark) while the participant was sitting in the MEG chamber. The hearing threshold for the TT is hard to determine directly, since it is confounded by the subjectively perceived internal tinnitus loudness. Therefore, the RT was used as a reference for an equal loudness matching in which the subject had to judge, if the TT had the same loudness as the RT. The intensity of the TT was adapted, until both stimuli were perceived as equally loud. This procedure was only conducted during the first measurement. The ascertained intensity ratio between RT and TT was used for all further MEG measurements. In each MEG session, 150 trials per condition (RT and TT) were presented in a randomized order in two blocks with a short break in between. The inter-stimulus interval was jittered between 2500 and 3500 ms. To keep subjects alert, they were allowed to watch a silent movie during MEG stimulation.

Tinnitus loudness assessment: Tinnitus loudness was judged before and after each MEG measurement via visual analog scales (VAS). The scale had a length of 10 cm and ranged from “tinnitus is absent” to “highest perceived tinnitus loudness ever” which was rated by marking a cross onto the scale line. In order to be sure that no Zwicker tone (a transient phantom sound) has appeared after notched music exposure [46], subjects were explicitly asked, if they perceived an additional tone directly after music exposure.

### MEG Recordings and Data Analysis

Continuous MEG data was recorded in a magnetically shielded room with a 275 channel whole head MEG system (Omega 275, CTF, VSM MedTech Ltd.) and digitally sampled at a rate of 600 Hz. The MEG pickup coils had an axial gradiometer configuration (2 cm diameter, 5 cm baseline, 2.2 cm distance between the sensor centres). Subjects were sitting in an upright position while their head was stabilized by cotton patches inside the MEG dewar. The stability of the head position during the MEG sessions was monitored by repeatedly measuring the magnetic fields of three head localization coils, set up inside the ear canals and upon the nasion (fiducial markers).

The current study investigates the impact of increased spectral energy contrasts onto inhibition-induced plasticity and therefore the concept of inhibition-induced plasticity. It additionally investigates parametrically a possible improvement of the effectiveness of the tailor-made notched music training. These research questions can be answered by analyzing the neural activity evoked by the TT. The RT is completely irrelevant for these research questions. However, we needed to present the RT to guarantee an adequate loudness matching and to keep paradigms between classical TMNM group [41] and ISEC-TMNM group equal. Furthermore, presentation of an additional tone during MEG recordings prevents habituation effects for the TT.For the sake of completeness, we provided the global field power evoked by the RT and TT separately in the supporting information (S3 Fig).

Data was preprocessed using Brain Electrical Source Analysis Software (BESA Research, version 6.0; Megis Software). First, an artefact correction was applied by calculating an independent component analysis (ICA) implemented in BESA [47] on the raw data. Based on the ICA results, contributions of eye blinks or heartbeats were reconstructed and subtracted from the raw MEG data. In a next step, continuous data was filtered between 1 and 45 Hz using a zero phase filter (transition steepness of 24 dB/octave) and afterwards segmented into epochs of 200 ms before stimulus onset to 1100 ms after stimulus onset. Data segments were baseline adjusted using a 100 ms interval before stimulus onset as reference and correspondingly averaged for each condition. Trials containing peak-to-peak amplitude differences greater than 3.0 pT were regarded as artefact contaminated and excluded from further analysis.

Neural sources underlying the auditory evoked fields were estimated using the least square minimum-norm estimation (L2-MNE, [48]) implemented in EMEGS [49] (www.emegs.org), a MATLAB based Software (The MathWorks, Natick, MA, USA). A spherical shell with 8 cm radius and 350 evenly distributed dipole locations, tangentially oriented to the spherical model, was used as a source model. The positions of the MEG sensors relative to the head were used to calculate individual lead-field matrices for each subject. The Tikhonov regularization parameter was set to λ = .1 for each subject and condition. To roughly represent the grey matter depth, a source shell radius of 87% of the individually fitted spherical conductivity model was chosen. A distributed source reconstruction of MEG signals does not reveal the precise location of neural generators; nevertheless, it results in a reasonably good approximation of cortical sources and corresponding assignment to larger cortical structures. The averaged magnetic field distributions and individual sensor positions for each subject were used to calculate topographies of source direction independent neural activities (i.e. the vector length of the estimated source activities at each position) for each time point, condition and individual subject.

A 3 x 2 x 2 mixed model Analysis of Variance (ANOVA) over all dipoles and time points with the within-subject factors *Day* (day 1, day 2, day 3) and *Session* (pre music exposure, post music exposure) and the between-subject factor *Group* (classical TMNM, ISEC-TMNM) was calculated for the L2-MNE values. A correction for multiple comparisons was applied on the results of interest of the ANOVA (main effect *Session* and interaction effect *Session* x *Group*) using a cluster-based permutation test for *F*-values [50]. The correction method included 1000 permutations and a minimal cluster size of five adjacent sources and five adjacent time points, with a Monte Carlo *P*-value of. 05. Significant spatiotemporal clusters in the N1m time interval (70–130 ms), which survived the cluster-based permutation test, for the main effect of *Session* and the interaction effect *Session x Group* were further analyzed with post-hoc *t*-test to disentangle the significant interaction effects.

For the case that significant effects were found in one hemisphere only, a lateralization analysis was conducted. This included the calculation of a lateral-symmetric spatiotemporal cluster by mirroring the significant dipole locations sagittally. An ANOVA for the mean neural activation of both clusters including the factor *Hemisphere* (right, left) was performed. Depending on the effect of interest, the ANOVA had either the factors *Session* x *Hemisphere*, or the factors *Session* x *Group* x *Hemisphere*, to calculate possible hemispheric differences.

Statistical analysis of behavioral data included a 2 x 2 mixed model ANOVA with the between subjects factor *Group* and the within subject factor *Session* for the mean VAS ratings before and after MEG measurements averaged across all three days.

## Results

### Sample Statistic

On average, groups did not differ significantly in age, TF, hearing loss at TF, maximum hearing loss ½ octaves above or below TF and VAS ratings on the first training day before music exposure. Table 1 shows detailed characteristics for each group individually and *p*-values of independent *t*-tests.

**Table 1:** Sample description differentiated between classical and ISEC-TMNM group.

	Mean (SD)		range	*p*-value
	Classical TMNM	ISEC-TMNM		
Age (years)	49.78 (13.41)	44.33 (7.60)	26–63	.31
TF (octaves)	2.26 (0.57)	1.76 (0.64)	0.85–3.09	.10
Hearing loss at TF (dB HL)	25.00 (14.36)	24.44 (14.46)	0–40	.94
Max. hearing loss ½ octave +/- TF (dB HL)	38.89 (17.28)	31.11 (15.16)	5–60	.33
VAS ratings on first session (cm)	5.38 (3.24)	6.36 (2.83)	1.60–10.00	.50

SD = standard deviation

### MEG Data

**Fig 3:**
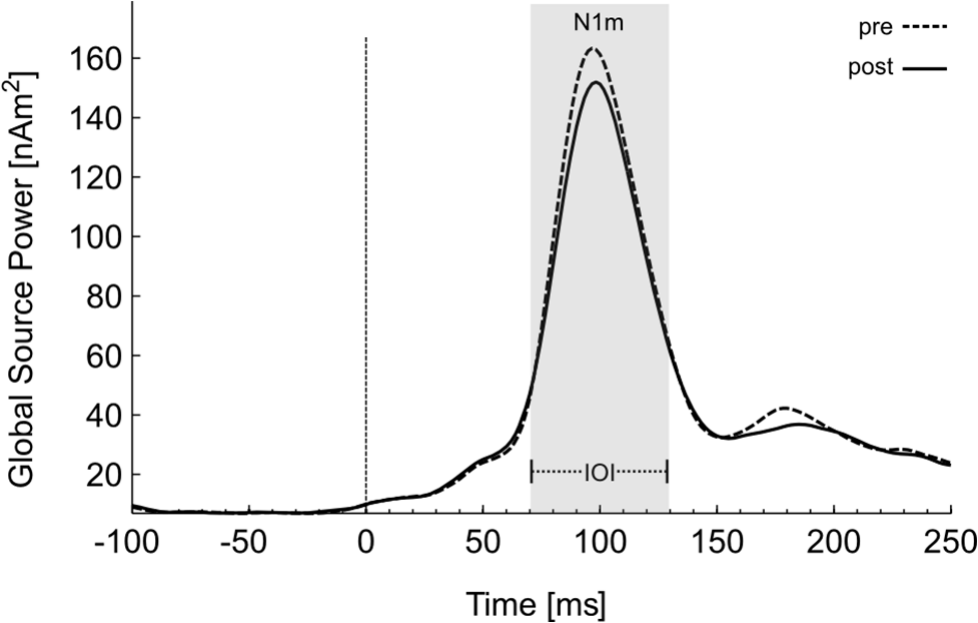
Global source power plot of the source waveforms averaged across all subjects. Neural activities evoked by the tinnitus tone pre (dotted line) and post (solid line) tailor-made notched music (TMNM) exposure. The a priori N1 time interval of interest (IOI) is highlighted in grey.

Fig 3 shows the global source power plot of the estimated neural activity evoked by the TF for all subjects (n = 18) and all test dipoles (n = 350) averaged across all days. A visual inspection of the N1m response in the predefined time window of 70–130 ms after stimulus onset reveals a reduction in the overall neural activity after music exposure.

The cluster-based permutation test for the *F*-values of the mixed model ANOVA Session (pre music exposure, post music exposure) x Group (classical TMNM, ISEC-TMNM) confirms the above mentioned observation by revealing a significant main effect for the factor *Session* (p <. 05, corrected) in five different regions (derived from S1 Dataset) for the a priori N1m time interval (70–130 ms).

**Fig 4:**
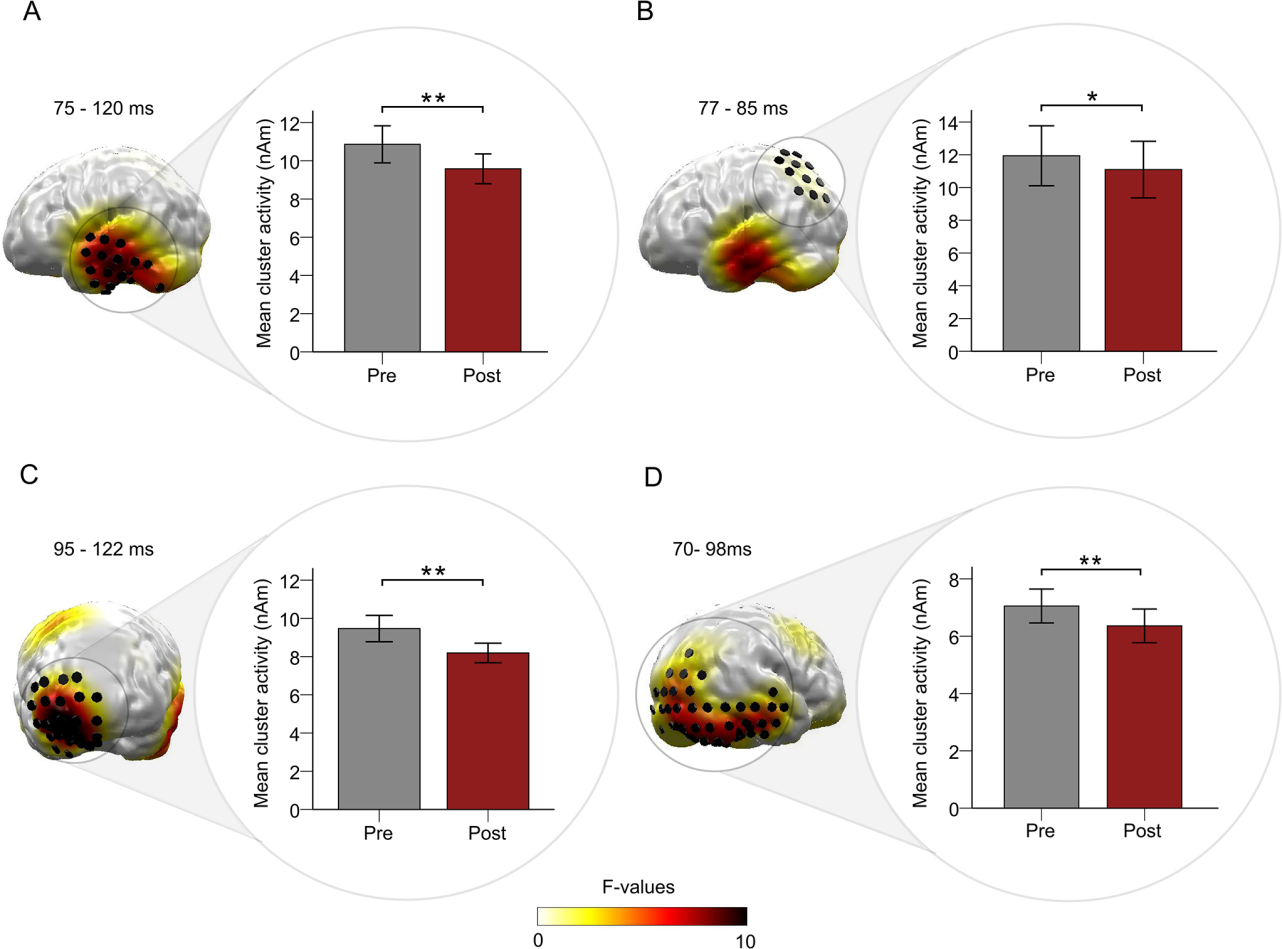
Results for the main effect *Session*. A: the right part depicts Statistical Parametric Maps of *F*-values for the main effect *Session* (pre vs. post tailor-made notched music (TMNM) exposure) in a temporal (B: parietal, C: orbitofrontal, D: occipital-temporal) cluster. Only spatiotemporal clusters which survived the cluster-based permutation test are colorized. Black cylinders reflect the source locations within the significant clusters. The right part framed with a circle disentangles the direction of the effect by depicting the mean activity evoked by the tinnitus tone before and after TMNM exposure within the significant temporal (B: parietal, C: orbitofrontal, D: occipital-temporal) cluster. Error bars denote one standard error of the mean. Stars denote significance-values of the *F*-test (** = *p* <. 01, * = *p* <. 05, *ns* = non-significant).

There was a significant reduction of neural activity evoked by the TF in a left temporal cluster (Fig 4A) for the time interval of 75–120 ms, *F* (1, 16) = 13.14, *p* = .002, η^2^ = .45. This effect could also be shown in a left parietal region (Fig 4B) between 77 and 85 ms, *F* (1, 16) = 5.62, *p* = .031, η^2^ = .26. Additionally, a right orbitofrontal cluster (Fig 4C) in the time window of 95–122 ms showed a significant reduction of TF evoked neural activity after music exposure (*F* (1, 16) = 15.91, *p* = .001, η^2^ = .50), as well as a right cerebral region with extents towards occipital and temporal areas (Fig 4D) for the time interval of 70–98 ms (*F* (1, 16) = 13.39, *p* = .002, η^2^ = .46). Furthermore, a right frontal region showed a significant main effect between 90 and 113 ms, *F* (1, 16) = 10.21, *p* = .006, η^2^ = .39. However, here TF evoked neural activity increased after music exposure.

To test for lateralization effects, the above mentioned data was reanalyzed taking the sagittally mirrored data into account. There was no significant interaction effect of *Session* x *Hemisphere* for the temporal, parietal and occipito-temporal clusters within the respective time intervals (all *F*-values < 1, all *p*-values >. 05) and thus lateralization effects for these spatiotemporal clusters are not further regarded. However, there was a significant interaction effect of *Session* x *Hemisphere* for the frontal (*F* (1, 16) = 6.78, *p* = 0.019) and the orbitofrontal cluster (*F* (1, 16) = 7.69, *p* = 0.014). Hence, both frontal clusters showed lateralization effects and only the right hemisphere should be further interpreted.

**Fig 5:**
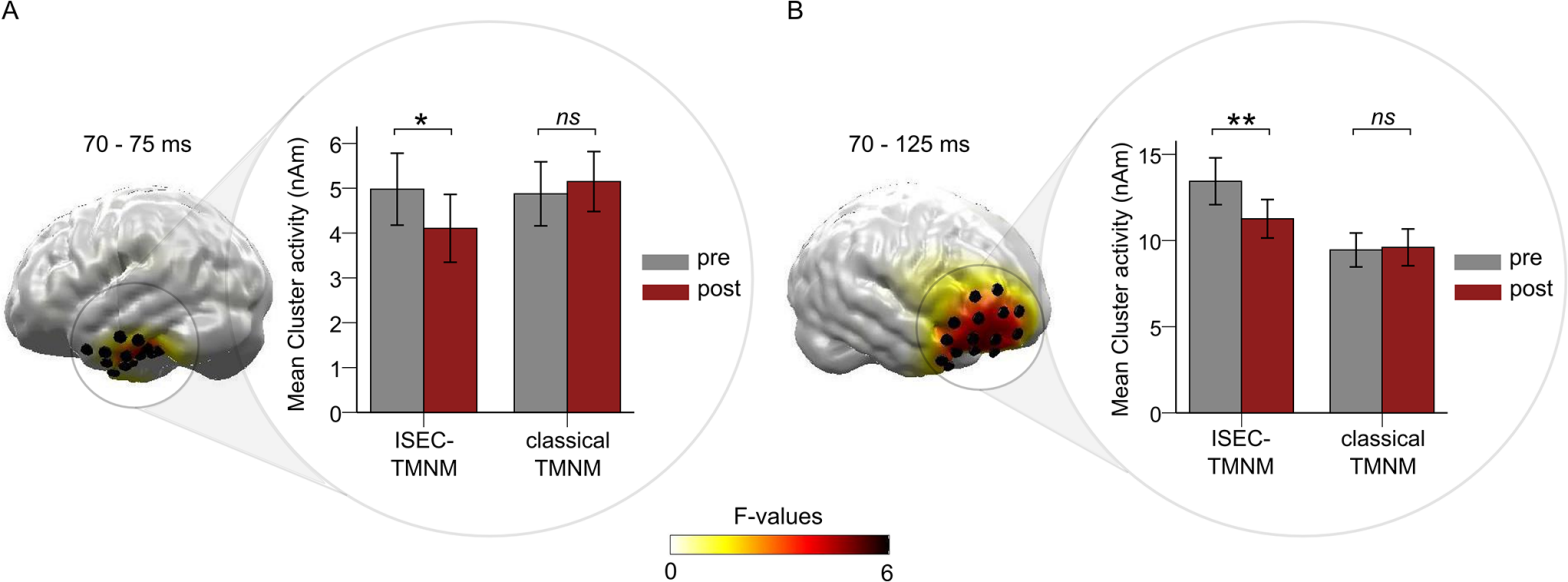
Results for the interaction effect *Session* x *Group*. A: left part depicts Statistical Parametric Maps of *F*-values for the interaction effect *Session* (pre vs. post tailor-made notched music (TMNM) exposure) x *Group* (increased spectral energy contrast (ISEC) vs. classical TMNM) in an inferior temporal (B: frontal) cluster. Only spatiotemporal clusters which survived the cluster-based permutation test are colorized. Black cylinders reflect the source locations within the significant clusters. The right part framed with a circle disentangles the direction of the interaction effect by depicting the mean activity evoked by the tinnitus tone before and after TMNM exposure within the significant inferior temporal (B: frontal) cluster for each group individually. Error bars denote one standard error of the mean. Stars denote significance values of pairwise *t*-tests disentangling the interaction effect (** = *p* <. 01, * = *p* <. 05, *ns* = non-significant).

Considering the type of TMNM, the cluster-based permutation test for the *F*-values of the interaction effect (*Session* x *Group*) revealed two significant spatiotemporal clusters in the predefined N1m time interval shown in Fig 5 (derived from S2 Dataset).

The first cluster was located in the left inferior temporal lobe (left panel of Fig 5A) between 70 and 75 ms, *F* (1, 16) = 6.77, *p* = .019, η^2^ = .30. There was a reduction of neural activity after music exposure in the ISEC-TMNM group, which was absent in the classical TMNM group (right part of Fig 5A). This observation was confirmed by pairwise *t*-tests resulting in a significant reduction of neural activity after music exposure in the ISEC-TMNM group (*t* (8) = 2.62, *p* = .031), but not in the classical TMNM group (*t* (8) = -0.95, *p* = .370).

A second significant spatiotemporal cluster for the interaction effect *Session* x *Group* was found in a frontal region (left part of Fig 5B) between 70 and 125 ms after stimulus onset, *F* (1, 16) = 10.11, *p* = .006, η^2^ = .39. Paired *t*-tests again resulted in a significant reduction of neural activity only for the ISEC-TMNM (*t* (8) = 3.521, *p* = .008) but not for the classical TMNM group (*t* (8) = -0.384, *p* = .711; right panel of Fig 5B).

Testing for lateralization effects by calculating the interaction effect for the factors *Session* x *Group* x *Hemisphere* (adding the sagittally mirrored data) resulted in non-significant differences between hemispheres for both clusters (all *F*-values < 1, all *p*-values >. 05). Hence a lateralization effect can be excluded for both regions.

Inspecting the global field power depicted in Fig 3 in more detail, reduced neural activity after TMNM exposure seems to be as well observable in the P2 time interval (150–250 ms). Since we did not define hypotheses for this time interval, we will only report significant effects with a significance level of p ≤. 01. Two significant spatiotemporal clusters for the interaction effect *Session x Group* were found in the P2 time interval (150–250 ms). The first cluster was located in a central-parietal region for the time interval 241–250 ms, *F* (1, 16) = 8.27, *p* = .01, η^2^ = .34. Paired *t*-tests resulted in a significant increment of neural activity in the classical TMNM group (*t* (8) = -3.15, *p* = .014), while the ISEC-TMNM group showed no significant differences. Additionally, a broad frontal regions revealed a significant interaction effect in the time interval between 190 and 248 ms, *F* (1, 16) = 9.97, *p* = .01, η^2^ = .38. Again we calculated paired *t*-tests to disentangle the interaction effect. While the ISEC-TMNM group showed a significant reduction of tinnitus-related neural activity after TMNM exposure (*t* (8) = 1.94, *p* = .045 (one-sided)), the classical TMNM group showed a significant increment of neural activity (*t* (8) = -2.76, *p* = .025). Results in the P2 time-window were reported for the sake of completeness and will not be discussed further, since this would extend the scope of this study.

### VAS Ratings

**Fig 6:**
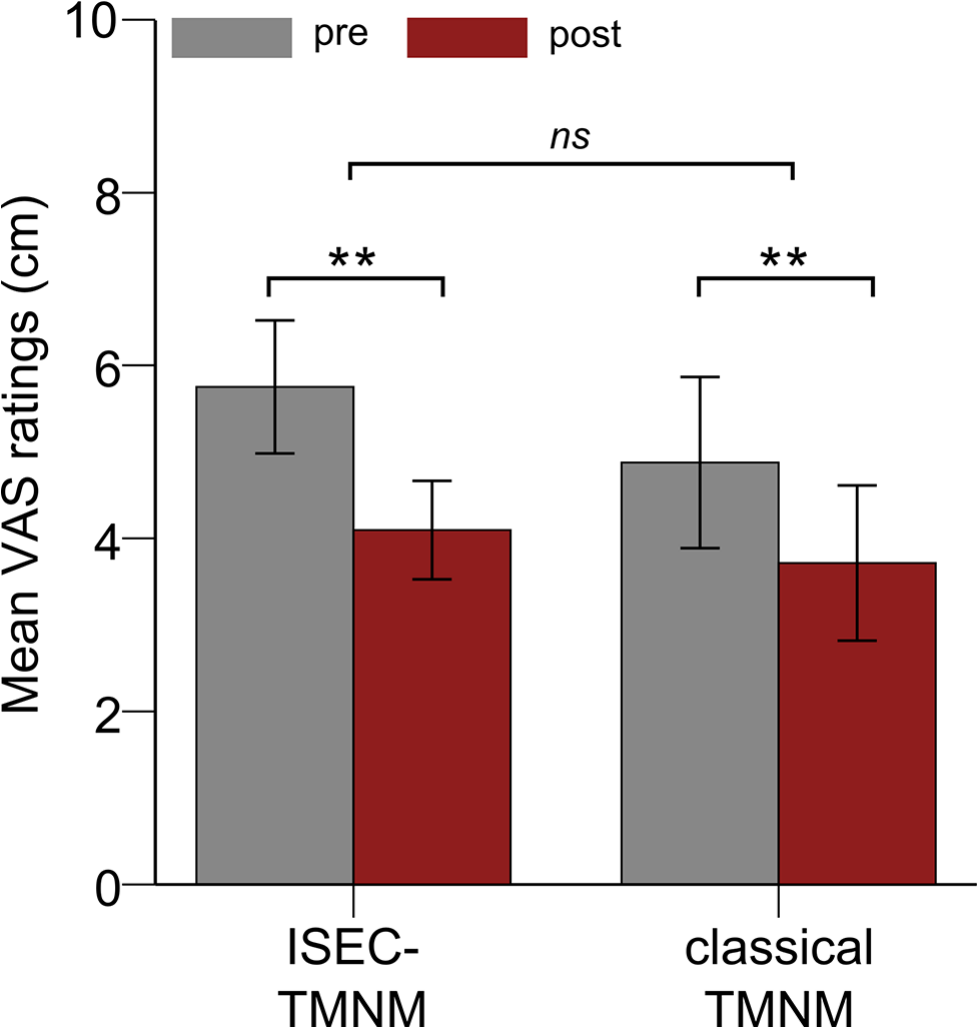
Tinnitus loudness ratings. Averaged ratings (across all three days) on a visual analog scale (VAS) of the subjectively perceived tinnitus loudness before (grey bar) and after (red bar) tailor-made notched music (TMNM) exposure for the increased spectral energy (ISEC) TMNM (left) and the classical TMNM (right) group. Error bars denote one standard error of the mean. Stars denote significance values of *F*-test depicting a significant main effect (** = *p* <. 01) of *Session* (pre vs. post music exposure) and a non-significant (*ns*) interaction effect *Session* x *Group*.

The mixed model ANOVA with the factors *Session* x *Group* for the VAS ratings revealed a significant main effect for the factor *Session*, *F* (1, 16) = 49.69, *p* <. 001, η^2^ = .76. As depicted in Fig 6 (derived from S3 Dataset), the subjectively perceived tinnitus loudness ratings were significantly reduced after TMNM exposure. Even though it seems that the ISEC-TMNM group had a slightly greater reduction of tinnitus loudness as compared to the classical TMNM group, there was no significant interaction effect between the factors *Session* and *Group*, *F* (1, 16) = 1.52, *p* = .235. Hence, the amount of tinnitus loudness reduction did not differ between both groups. No participant reported to perceive a Zwicker-tone after TMNM exposure.

## Discussion

Plasticity is defined as the dynamical adaptation of our nervous system due to experience [1–3]. We could demonstrate that listening to three hours of TMNM on three consecutive days resulted in a reduction of tinnitus related neural activity. Thus, the functional deafferentation of neurons coding the TF during TMNM exposure resulted in fast evolving plastic changes. This inhibition-induced plasticity was observed in all regions of the so called tinnitus network comprising temporal, parietal and right frontal [42–44] regions. Additionally, there was a significant increase of neural activity after music exposure in a right dorsolateral prefrontal region most presumably reflecting perceptual learning without training [41, 51–53]. We doubled our sample size as compared to Stein and Engell et al. [41] and still found the same rapidly evolving reduction of tinnitus related activity in all areas of the tinnitus network. Therefore, the effects of inhibition-induced plasticity can be assumed to be robust.

Another spatiotemporal cluster revealing a significant reduction of tinnitus-related neural activity was located in a cerebral region with extents into occipital and temporal areas. We assume this cluster as reflecting activity in the cerebellum, which extends to neighboring areas as consequence of the L2MNE bias towards distributed and superficial sources (i.e. deeper and more focal sources will be estimated as more superficial and more distributed [54,55]). Interestingly, the cerebellum could be identified as being involved in neural pain [56,57] and phantom pain processing [58] matching the view that tinnitus is often referred to as a phantom pain sensation [6]. Indeed, recent studies revealed increased cerebellar activity during tinnitus perception [59–61]. A reduction of tinnitus related neural activity after TMNM exposure in the cerebellum thus argues for a reduction in tinnitus processing due to inhibition-induced plasticity. This effect of reduced cerebellar activity after therapy was already observed in the *classical TMNM group* in a previous study by Stein and Engell et al. (2014) [41], though not interpreted further as the cerebellum had at that time not been an a priori defined region of interest. Since this effect is now still observed, even after increasing the sample to n = 18, it seems to be involved in tinnitus processing and inhibition-induced plasticity via TMNM. However, further research focusing on the role of the cerebellum in tinnitus perception needs to be done.

The main aim of the current study was to investigate whether inhibition-induced plasticity is modifiable by varying spectral energy contrasts in music. More precisely we asked the question if amplifying the edge frequency bands around the notch results in increased inhibition of neurons coding the TF as compared to classical TMNM. We found two regions, both part of the tinnitus network, which showed a reduction of neural activity evoked by TF after TMNM exposure for the ISEC-TMNM group only. This outcome argues for a superior effect of amplified edge frequency bands around the notch on inhibition-induced plasticity in addition to the general inhibition-induced plasticity effect in the tinnitus network.

One significant spatiotemporal cluster was located in the inferior temporal lobe, reflecting inhibition in the auditory cortex due to increased spectral energy contrasts in TMNM. These results confirm previous findings that lateral inhibition is modifiable in the auditory cortex [32,33], especially when varying the spectral energy contrasts [40]. Therefore, basic effects of lateral inhibition found in normal hearing subjects seem to be transferable to effects of inhibition-induced plasticity in participants suffering from chronic tinnitus.

Interestingly, the second significant spatiotemporal cluster revealing a greater inhibition of tinnitus related neural activity for the ISEC-TMNM group was found in a right prefrontal cortical region. Most research investigating modulation of inhibition via spectral energy contrasts in sounds so far focused on the auditory cortex [32,33,37,40]. However, as mentioned above, tinnitus related activity occurs in a distributed tinnitus network, comprising not only auditory sensory, but also parietal and frontal cortex regions [42–44]. Furthermore, the frontal cortex is known to be involved in the processing of emotional stimuli [62,63] and in pain [57] as well as phantom pain perception [58]. Since TMNM induces inhibition onto tinnitus related neurons, it seems reasonable that a reduction of tinnitus related neural activity due to inhibition-induced plasticity and thus a suggested reduction of emotional processing or phantom perception is observed in right frontal areas.

Our study aimed at testing the concept of inhibition-induced plasticity. We argued, following the theory of lateral inhibition in the auditory cortex, that neurons coding the edge frequency bands around the notch should have the greatest influence on lateral inhibition of neurons coding the center frequency of the notch. Therefore, the ISEC-TMNM group should have a superior inhibition-induced plasticity as compared to the classical TMNM group. In addition to the reduction of TF related neural activity in the tinnitus network after TMNM exposure, we also found an improvement of inhibition-induced plasticity within the N1 time window in two regions of this network due to spectral energy contrast amplification. Thus, the current results confirm our hypothesis and, accordingly, the concept of inhibition-induced plasticity.

The second aim of this study was to find a parameter which possibly improves TMNMT [30]. Behavioral data revealed a significant overall reduction of tinnitus loudness after TMNM exposure in both groups. However, there was no effect of TMNM type on the loudness reduction. We argued that inhibition-induced plasticity should be modulated on a neural as well as a behavioral level. Therefore, we assumed that the subjectively perceived tinnitus loudness should be stronger reduced in the ISEC-TMNM group after music exposure as compared to the classical TMNM group. This hypothesis could not be confirmed in our results, since we could only show an effect of spectral energy contrasts in TMNM on a neural level, but not on a behavioral one.

One reason for the missing modulation of inhibition-induced plasticity due to spectral energy contrast variation on subjective tinnitus loudness might be due to the low sensitivity of the VAS. Even if the VAS seems to be a valid and reliable instrument for tinnitus loudness change measurements [64], it might not have been sensitive enough to measure small change effects. This might especially be the case with regard to the rather small sample size and therefore reduced power for behavioral effects. Also placebo or social expectancy effects in the subjectively perceived tinnitus loudness ratings cannot be excluded. Another possibility might be the relatively short exposure time to TMNM since plasticity often is first observed on a neural basis and only later manifested in modulated behavior [65]. Therefore, these music modifications should be investigated for a prolonged treatment period to test for tinnitus relieving effects.

Another limitation of this study is that we could not analyze the exact loudness level which our participants chose to listen to TMNM. However, we assume that the loudness level should be balanced between subjects. Our participants had to listen to TMNM in a comfortable loudness so that they were able to enjoy the music. Since hearing loss values and possible recruitment mechanisms varied between subjects, it was not possible to present the music with equal loudness for all subjects. If the loudness level would have differed between groups, it should have been lower for the ISEC-TMNM group, since here, the edge frequency bands were amplified at about 20 dB. On the other hand, we found greater inhibition-induced plasticity in this group. So if the comfortable loudness in the ISEC-TMNM group was lower as compared to classical TMNM, than increased spectral energy contrasts would even have greater influence, since lateral inhibition would have been induced, even though there was less overall energy in the music. This would be in line with our hypotheses. Overall, the current results confirmed the involvement of a distributed tinnitus neural network in inhibition-induced plasticity by TMNM exposure. We were able to demonstrate an improvement of inhibition-induced plasticity in people suffering from chronic tinnitus as a consequence of spectral energy contrasts. This improvement was found in temporal as well as frontal cortical regions, thereby confirming the concept of inhibition-induced plasticity.

## Supporting Information

S1 Dataset Dataset of the tinnitus related neural activity in five significant clusters for the main effect *Session*. Mean activity of significant clusters for the main effect *Session* (pre TMNM exposure vs. post TMNM exposure) which survived the applied cluster-based permutation test for all subjects (group: 0 = classical TMNM; 1 = increased spectral energy contrast TMNM). Significant clusters were located in a left temporal (left_temp), a left parietal (left_par), a right orbitofrontal (right_orb_front), a right cerebral region with extents towards occipital and temporal areas (right_cerebral) and a right frontal region (right frontal).
(XLSX)

S2 Dataset Dataset of the tinnitus related neural activity in five significant clusters for the interaction effect *Session* x *Group*. Mean activity of significant clusters for the interaction effect *Session* (pre TMNM exposure vs. post TMNM exposure) x *Group* (classical TMNM (coded with ‘0’) vs. increased spectral energy contrast TMNM (coded with ‘1’)) which survived the applied cluster-based permutation test for all subjects. Significant clusters were located in a left inferior temporal (left_temp) and a right frontal region (right_frontal).
(XLSX)

S3 Dataset Dataset of the visual analog scale ratings. Mean ratings of the subjectively perceived tinnitus loudness averaged for each session and across all three days before (pre) and after (post) TMNM exposure. Scale ranged from ‘tinnitus is absent’ (0.00 cm) to ‘loudest tinnitus ever perceived’ (10.00 cm). ‘Group’ is coded as follows: 0 = classical TMNM; 1 = increased spectral energy contrast TMNM.
(XLSX)

S1 Fig Source waveforms of significant sources for the interaction effect Session x Group in the temporal region. Continuous lines show the source power evoked by the tinnitus tone before tailor-made notched music (TMNM) exposure; dashed lines indicate the tinnitus related source power after TMNM exposure. Grey lines reflect the source power of the classical TMNM group while the black lines indicate the source power of the ISEC-TMNM group. The averaged source waveforms depict only the activity of temporal sources, which showed a significant interaction effect *Session* x **Group** and survived the applied cluster-based permutation test.
(TIF)

S2 Fig Source waveforms of significant sources for the interaction effect Session x Group in the frontal region. Continuous lines show the source power evoked by the tinnitus tone before tailor-made notched music (TMNM) exposure; dashed lines indicate the tinnitus related source power after TMNM exposure. Grey lines reflect the source power of the classical TMNM group while the black lines indicate the source power of the ISEC-TMNM group. The averaged source waveforms depict only the activity of frontal sources, which showed a significant interaction effect *Session* x *Group* and survived the applied cluster-based permutation test.
(TIF)

S3 Fig Global Field Power of the activity of the neural sources evoked by the reference and the tinnitus tone. Continuous lines show the global field power of measurements before exposure to tailor-made notched music (TMNM, pre); dashed lines indicate the global field power after TMNM exposure (post). Black lines reflect neural activity evoked by the reference tone, while grey lines indicate tinnitus related neural activity. Since the reference tone had a far lower carrier frequency (500 Hz) as compared to the tinnitus frequency (ranging from 1500–8500 Hz), the global field power for the lower tone is overall greater than for the tinnitus tone. Comparisons of pre and post measures indicate, that tinnitus related neural activity decreased after TMNM exposure, while neural activity evoked by the reference tone remained the same.
(TIF)
