# Supplementary figures and images for: Enhancing Inhibition-Induced Plasticity in Tinnitus – Spectral Energy Contrasts in Tailor-Made Notched Music Matter
Source: PLoS One. 2015 May 7;10(5):e0126494. doi: 10.1371/journal.pone.0126494 (PMC4423974; doi:10.1371/journal.pone.0126494)

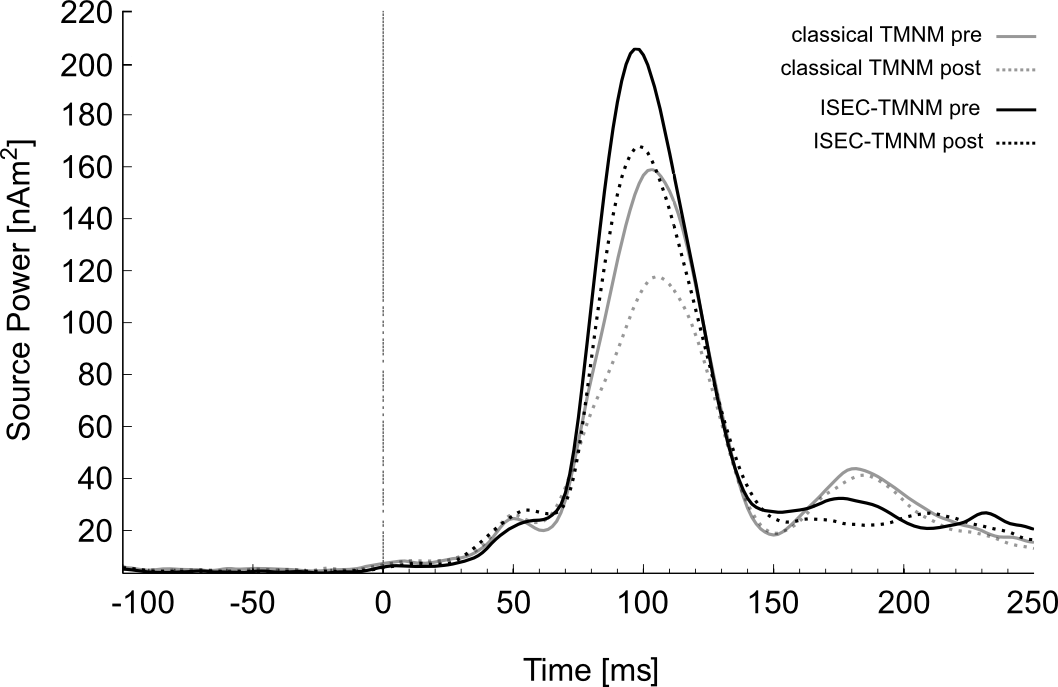

Supplement: S1 Fig — Continuous lines show the source power evoked by the tinnitus tone before tailor-made notched music (TMNM) exposure; dashed lines indicate the tinnitus related source power after TMNM exposure. Grey lines reflect the source power of the classical TMNM group while the black lines indicate the source power of the ISEC-TMNM group. The averaged source waveforms depict only the activity of temporal sources, which showed a significant interaction effect Session x Group and survived the applied cluster-based permutation test. (TIF) [file pone.0126494.s004.tif]

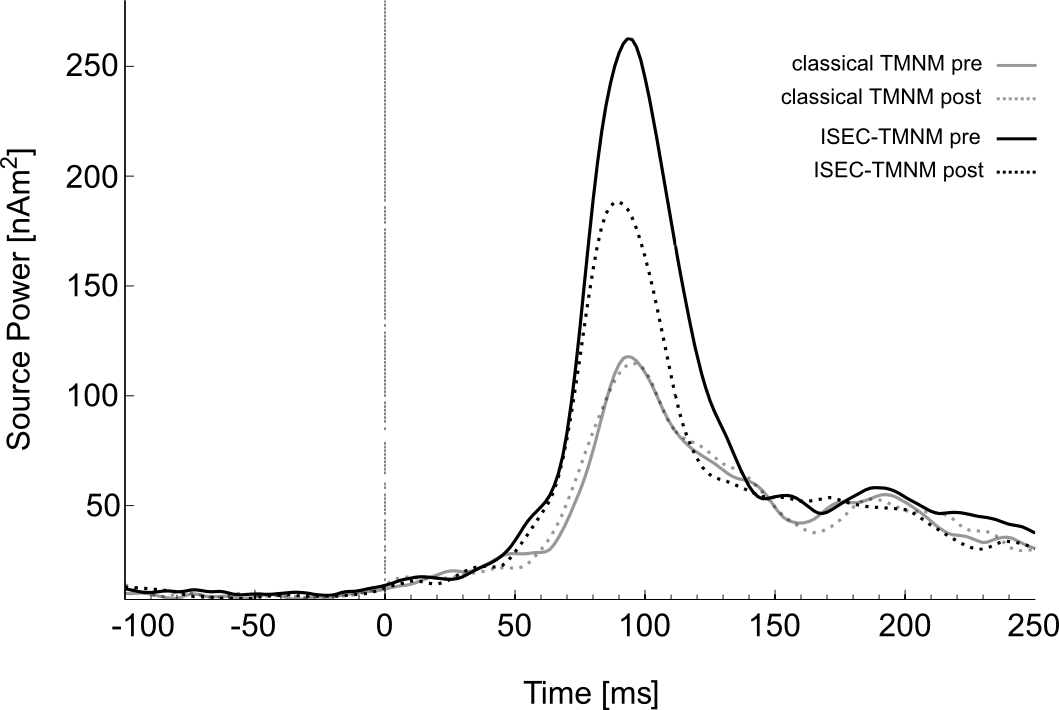

Supplement: S2 Fig — Continuous lines show the source power evoked by the tinnitus tone before tailor-made notched music (TMNM) exposure; dashed lines indicate the tinnitus related source power after TMNM exposure. Grey lines reflect the source power of the classical TMNM group while the black lines indicate the source power of the ISEC-TMNM group. The averaged source waveforms depict only the activity of frontal sources, which showed a significant interaction effect Session x Group and survived the applied cluster-based permutation test. (TIF) [file pone.0126494.s005.tif]

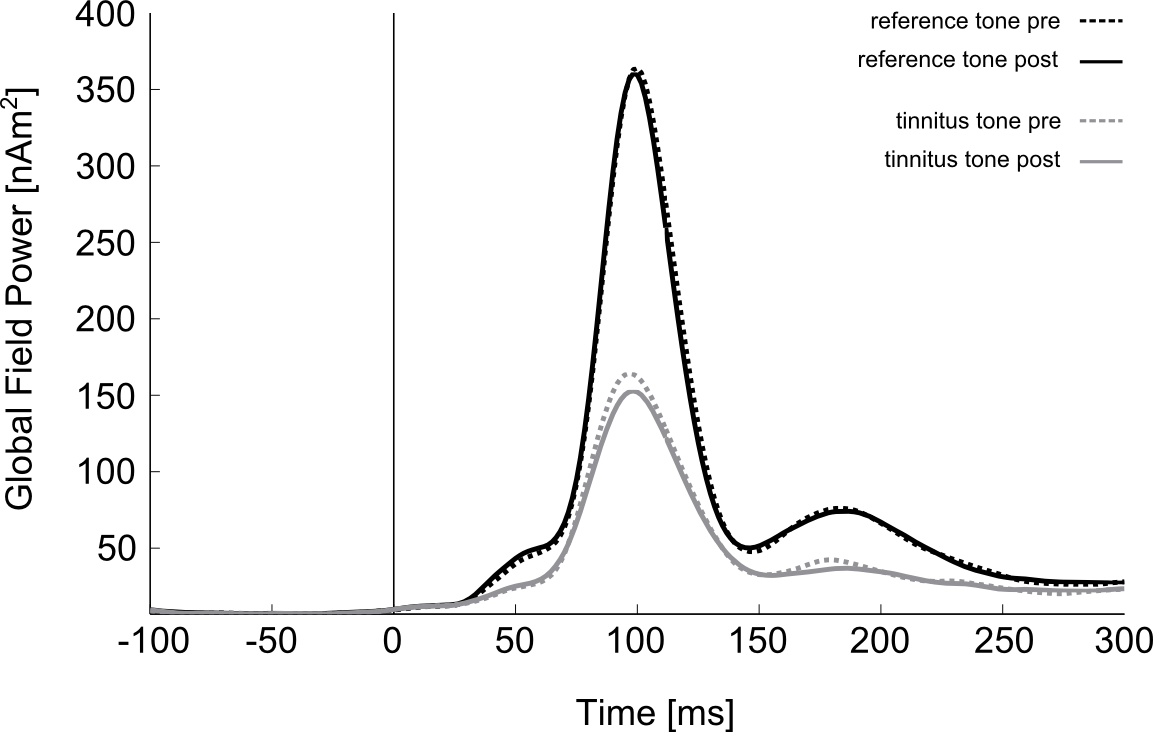

Supplement: S3 Fig — Continuous lines show the global field power of measurements before exposure to tailor-made notched music (TMNM, pre); dashed lines indicate the global field power after TMNM exposure (post). Black lines reflect neural activity evoked by the reference tone, while grey lines indicate tinnitus related neural activity. Since the reference tone had a far lower carrier frequency (500 Hz) as compared to the tinnitus frequency (ranging from 1500–8500 Hz), the global field power for the lower tone is overall greater than for the tinnitus tone. Comparisons of pre and post measures indicate, that tinnitus related neural activity decreased after TMNM exposure, while neural activity evoked by the reference tone remained the same. (TIF) [file pone.0126494.s006.tif]
